# Outcomes of Non-vascularized Fibular Grafts in Proximal Humerus Aneurysmal Bone Cysts

**DOI:** 10.7759/cureus.66428

**Published:** 2024-08-08

**Authors:** Ahmed Ismail, Ahmed Ashour, Ehab Alieldin, Ahmed T Ashour, Ahmed Abouelnaga, Mahmoud Salama, Ahmed M Attia, Islam Sarhan

**Affiliations:** 1 Orthopaedics, Bradford Royal Infirmary, Bradford, GBR; 2 Trauma and Orthopaedics, Queen Elizabeth Hospital, Birmingham, GBR; 3 Trauma and Orthopaedics, The Royal London Hospital, London, GBR; 4 Trauma and Orthopaedics, Elhadara University Hospital, Alexandria, EGY; 5 Trauma and Orthopaedics, Manchester Royal Infirmity, Manchester, GBR; 6 Trauma and Orthopaedics, University Hospitals of Alexandria, Alexandria, EGY; 7 Trauma and Orthopaedics, Barts Health National Health Service (NHS) Trust, London, GBR; 8 Trauma and Orthopedics, Alexandria University, Alexandria, EGY; 9 Trauma and Orthopaedics, University Hospitals of Derby and Burton National Health Service (NHS) Foundation Trust, Derby, GBR

**Keywords:** non-vascularised fibular graft, proximal humerus with curettage, clinical orthopaedics, aneurysmal bone cysts, onco-surgery

## Abstract

Introduction

This study focuses on the management of primary aneurysmal bone cysts (ABCs) through comprehensive curettage, hydrogen peroxide lavage, and non-vascularized strut fibular grafting.

Methods

The research encompassed 20 cases, predominantly males (80%), with an average age of 11.35 years. Patient assessment involved thorough history-taking, clinical examinations, and radiological evaluations, including plain radiographs, CT scans, and MRI. The study evaluated healing, bone consolidation, and complications, with patients assessed using the Musculoskeletal Tumor Society (MSTS) upper limb score.

Results and discussion

The results demonstrated a mean MSTS score of 91.55%, indicating favorable outcomes compared to prior studies. The utilization of non-vascularized autogenous fibular grafts offered effective mechanical stabilization and facilitated an early return to normal function, even prior to complete cavity filling. Our research underscores the efficacy of this treatment approach for primary ABC, particularly in achieving satisfactory functional outcomes. Moreover, the findings contribute to the understanding of optimal management strategies for ABC, considering factors such as patient age, lesion location, vascularity, and size.

Conclusion

The study advocates for the adoption of thorough curettage, hydrogen peroxide lavage, and non-vascularized strut fibular grafting as a reliable treatment modality for primary ABC. This approach highlights its potential to enhance patient outcomes and functional recovery.

## Introduction

Aneurysmal bone cysts (ABCs) frequently occur in the upper humerus, predominantly in the proximal humeral metaphysis or shaft, and are prevalent benign tumors among children, adolescents, and young adults [1]. Pathological fractures occasionally serve as the initial indication of this condition [1], often discovered incidentally during routine radiographic assessments.

Treatment typically involves curettage with or without bone grafting, although this approach carries a notable risk of complications such as recurrence, refracture, and pseudarthrosis [2-7]. Adjunctive therapies like concentrated ethanol or hydrogen peroxide have been employed to reduce local recurrence rates [8, 9].

Current treatment modalities for ABCs also encompass techniques such as bone marrow or bone matrix injection [10-14], curettage with or without bone grafting [15-17], fixation using cannulated screws [18-20], or combinations thereof [20].

Bone grafting options include autologous and allograft bone. Autologous bone grafts offer high success rates, minimal infection risk, and excellent histocompatibility [21- 23]. Allografts, lacking the same growth potential as autografts, present a higher infection risk, limiting their utility in cyst filling, particularly in pediatric cases [10, 11]. Calcium phosphate ceramics serve as osteoconductive bone void fillers [21, 22].

Chemical adjuvants such as phenol (3%), ethanol, hydrogen peroxide, and alcohol are utilized to augment curettage in aggressive benign bone lesions, effectively lowering recurrence rates and reducing toxicity compared to other adjuvants [22, 23].

Surgical indications include painful swelling, pathological fractures, and restricted range of motion. The widely accepted surgical approach involves curettage with or without bone grafting and adjunctive therapies.

This retrospective study aims to report on the clinical, radiographic, and functional outcomes of 20 patients with ABCs of the proximal humerus treated with meticulous curettage, hydrogen peroxide lavage, and impaction of autologous non-vascularized fibular grafts.

## Materials and methods

Study design and participants

This retrospective study included 20 patients aged eight to 18 years, predominantly males (80%), between January 2019 and December 2021 with ABCs in the proximal humerus. Patients' preoperative, operative, and postoperative data with close follow-up for up to six months were reviewed. Patients were treated at El-Hadara University Hospital in Alexandria, Egypt. The research question was: What was the outcome of treating proximal humeral aneurysmal bone cysts with a non-vascularized fibular graft?

The inclusion criteria were patients with lesions whose length was twice or more than the narrowest cortical diameter of the humerus. The exclusion criteria were secondary ABCs (excluded by age group, clinical history, and radiography) and small ABCs that were mechanically stable after curettage.

Data collection and evaluation

Radiological and Histopathological Evaluation

Graft positioning and healing were assessed using immediate postoperative radiographs, followed by evaluations at eight weeks and six months postoperatively. X-rays were taken in anteroposterior and lateral views. The cyst’s length-to-circumference ratio was assessed using the longitudinal axis of the cyst divided by the narrowest cortical diameter of the humerus at the lesion site. Histopathological evaluation postoperatively confirmed 100% of cases as ABCs. Biopsies were taken from all around the lesion to avoid missing any coexisting pathology.

Treatment Protocol

Twenty patients with proximal humerus ABCs were treated with thorough curettage, hydrogen peroxide lavage, and autologous non-vascularized fibular graft impaction using a trans-deltoid approach.

The postoperative regimen included several stages. On the first day, a postoperative plain radiograph was taken, and the arm was placed in an arm sling. Four weeks postoperatively, the arm sling was removed, and patients began passive movements of the shoulder and elbow. Eight weeks postoperatively, a follow-up radiograph was conducted to assess healing and consolidation, and patients were instructed on active movements of the shoulder. Six months postoperatively, another follow-up radiograph was taken to assess healing, recurrence probability, and cyst filling completion. The Musculoskeletal Tumor Society Score (MSTS) was then interpreted [23]. This score was utilized to assess functional outcomes according to six criteria (Table 1).

**Table 1:** MSTS score assessment results The data presented in this table are adapted from the Musculoskeletal Tumor Society (MSTS), with permission obtained from Deborah Meyer, Society Manager of the Musculoskeletal Tumor Society.

Score	0	1	2	3	4	5
Pain	Severe/ disabling	Moderate/ disabling	Moderate	Modest/non-disabling	Intermediate	No pain
Function	Total restriction	Partial restriction	Moderate	Recreational restriction	Intermediate	No restrictions
Emotional	Dislikes	Accepts	Intermediate	Satisfied	Intermediate	Enthused
Hand positioning	Non	Not above waist	Moderate	Not above shoulder or no pronation-supination	Intermediate	Unlimited
Manual Dexterity	Cannot grasp	Cannot pinch	Moderate	Loss of fine movements	Intermediate	Unlimited
Lifting ability	Cannot help	Helping only	Moderate	Limited	Intermediate	Normal load

Statistical analysis

Data were analyzed using IBM SPSS Statistics for Windows, version 20.0 (IBM Corp., Armonk, NY). Qualitative data were presented as numbers and percentages. Quantitative data were presented using range (minimum and maximum), mean, standard deviation, median, and interquartile range (IQR). The Kolmogorov-Smirnov test confirmed normal distribution. Statistical significance was set at the 5% level. The statistical tests used included the Student's t-test for the normally distributed quantitative variables to juxtapose between two studied groups in addition to the F-test (ANOVA) for normally divided quantitative variables to correlate between more than two groups, along with the Fisher's exact test used to determine if there are nonrandom associations between two categorical variables in a 2x2 contingency table. It is especially useful when sample sizes are small. A p-value of 0.005 or less was considered statistically significant.

## Results

Results according to the MSTS score percentage

**Figure 1:**
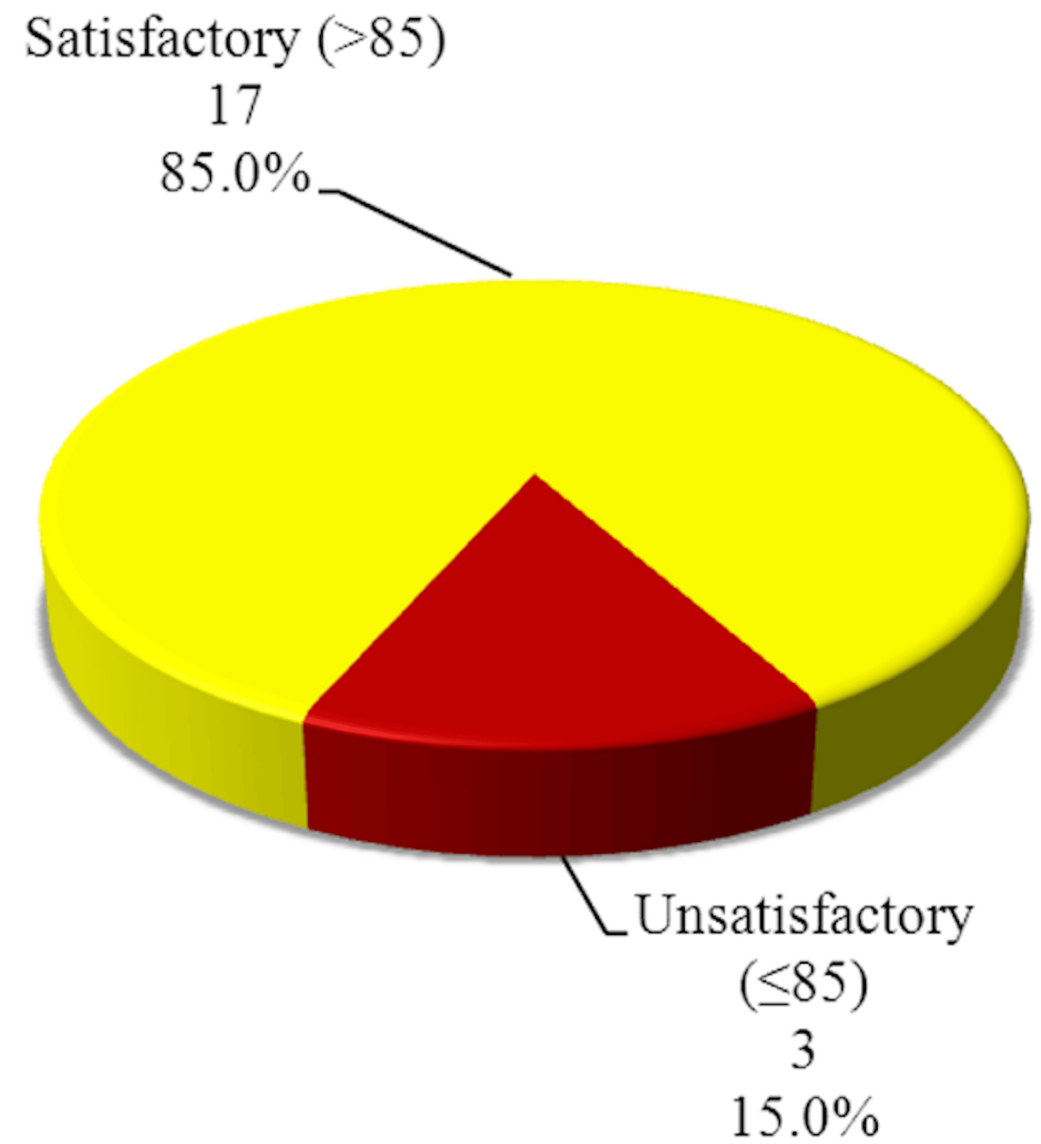
Descriptive analysis of the studied cases according to percentage

The MSTS upper limb score percentage, while subjective, highlights a high overall success rate, with the majority of patients achieving satisfactory outcomes. The highest score was 100%, and the lowest score was 67%. The mean percentage of the total MSTS score in this study was 91.55%. While no scoring system from MSTS guides further action, we hypothesized in this study, that a score of more than 85% (17 patients) would be considered satisfactory, while those with a score of 85 or less (three patients) would be considered unsatisfactory (Figure 1).

Distribution according to pain tolerance (subjective)

Pain management post-surgery was notably effective, as the majority of patients reported no pain or only intermediate pain that improved over time, which is superior to leaving the cyst alone, especially those who presented with a fracture and left to heal. Fifteen patients (75%) had no pain eight weeks postoperatively, and five patients (25%) had intermediate pain with no interference with function but improved subsequently (Table 2).

**Table 2:** Distribution of the studied cases according to pain Min: minimum; Max: maximum; SD: standard deviation; IQR: interquartile range

Pain	No. (%)
No pain	15 (75.0)
Intermediate	5 (25.0)
Modest/non-disabling	0 (0.0)
Intermediate	0 (0.0)
Moderate/disabling	0 (0.0)
Severe/disabling	0 (0.0)
Total	20 (100.0)
Min. – Max.	4.0 – 5.0
Mean ± SD.	4.75 ± 0.44
Median (IQR)	5.0 (4.50 – 5.0)

Distribution according to functional rehabilitation

Functional rehabilitation outcomes were promising, with most patients experiencing minimal to no restrictions, which underscores the effectiveness of the graft in restoring limb function almost immediately postoperatively, which is the main goal of our study. Fourteen patients (70%) had no restriction of function, and six patients had an intermediate restriction but with no impact on quality of life (Table 3).

**Table 3:** Distribution according to functional rehabilitation Min: minimum; Max: maximum; SD: standard deviation; IQR: interquartile range

Function	No. (%)
No restrictions	14 (70.0)
Intermediate	6 (30.0)
Recreational restriction	0 (0.0)
Intermediate	0 (0.0)
Partial restriction	0 (0.0)
Total restriction	0 (0.0)
Total	20 (100.0)
Min. – Max.	4.0 – 5.0
Mean ± SD.	4.70 ± 0.47
Median (IQR)	5.0 (4.0 – 5.0)

Distribution according to emotional satisfaction

While emotional satisfaction is inherently subjective, it provides valuable insights into postoperative outcomes, particularly in the context of patient and parental concerns about additional scarring from fibular graft harvesting. Overall, patients' emotional satisfaction was found to be superior in those who underwent fibular grafting compared to those who received iliac bone-filling grafts. This higher satisfaction is likely due to several factors: the absence of a need for a second surgery to remove hardware, the rapid healing of the fibular graft site, and the immediate weight-bearing capability postoperatively. These advantages contribute to a more positive overall patient experience and emphasize the clinical benefits of using non-vascularized autogenous fibular grafts in the treatment of proximal humeral ABCs. Eleven patients (55%) were enthused and satisfied with the results with no complaints (excellent results); seven patients had an intermediate response (good results); and two patients (10%) were satisfied with minor complaints and improved subsequently (Table 4).

**Table 4:** Distribution of the studied cases according to emotional satisfaction Min: minimum; Max: maximum; SD: standard deviation; IQR: interquartile range

Emotion	No. (%)
Enthused	11 (55.0)
Intermediate	7 (35.0)
Satisfied	2 (10.0)
Intermediate	0 (0.0)
Accepts	0 (0.0)
Dislikes	0 (0.0)
Total	20 (100.0)
Min. – Max.	3.0 – 5.0
Mean ± SD.	4.45 ± 0.69
Median (IQR)	5.0 (4.0 – 5.0)

Distribution according to hand positioning

Hand positioning directly correlates with a patient's ability to perform essential daily tasks such as eating, dressing, and personal hygiene. It provides an objective measure of functional capability, allowing clinicians to observe and quantify the range of motion, making it a reliable indicator of postoperative recovery. Twelve patients (60%) had unlimited hand movement and positioning, seven patients (35%) had intermediate movements but with no restrictions to quality of life (good result), and one patient (5%) had no movement above shoulder level (90 degrees) (fair result) but improved subsequently (Table 5).

**Table 5:** Distribution of the studied cases according to hand positioning

Hand positioning	No. (%)
Unlimited	12 (60.0)
Intermediate	7 (35.0)
Not above the shoulder	1 (5.0)
Intermediate	0 (0.0)
Not above the waist	0 (0.0)
None	0 (0.0)
Total	20 (100.0)

Distribution according to manual dexterity

Manual dexterity evaluates fine motor skills and the ability to perform precise movements, which are essential for tasks such as writing, buttoning clothes, and handling small objects. It reflects the intricate coordination between muscles, tendons, and nerves in the upper limb. Twelve patients (60%) had unlimited manual dexterity (excellent results), while eight patients (40%) had intermediate dexterity but with no loss of fine movements (good results) but improved subsequently (Table 6).

**Table 6:** Distribution of the studied cases according to manual dexterity Min: minimum; Max: maximum; SD: standard deviation; IQR: interquartile range

Manual dexterity	No. (%)
Unlimited	12 (60.0)
Intermediate	8 (40.0)
Loss of fine movements	0 (0.0)
Intermediate	0 (0.0)
Cannot pinch	0 (0.0)
Cannot grasp	0 (0.0)
Total	20 (100.0)
Min. – Max.	4.0 –5.0
Mean ± SD.	4.60 ± 0.50
Median (IQR)	5.0 (4.0 – 5.0)

Distribution according to lifting ability

Lifting ability assesses the strength and endurance of the upper limb, crucial for tasks that involve carrying or lifting objects, such as grocery bags or children. It is an indicator of the overall muscle strength and functional capacity of the limb. Nine patients (45%) had complete lifting ability (excellent prognosis), and 10 patients (50%) had intermediate lifting ability (good prognosis) compared to the other limb after eight weeks but with no interference with quality of life. One patient (5%) had limited lifting ability (fair result) but improved subsequently (Table 7).

**Table 7:** Distribution of the studied cases according to lifting ability Min: minimum; Max: maximum; SD: standard deviation; IQR: interquartile range

Lifting ability	No. (%)
Normal load	9 (45.0)
Intermediate	10 (50.0)
Limited	1 (5.0)
Intermediate	0 (0.0)
Helping only	0 (0.0)
Cannot help	0 (0.0)
Total	20 (100.0)
Min. – Max.	3.0 – 5.0
Mean ± SD.	4.40 ± 0.60
Median (IQR)	4.0 (4.0 – 5.0)

Relation between healing time and recurrence

**Figure 2:**
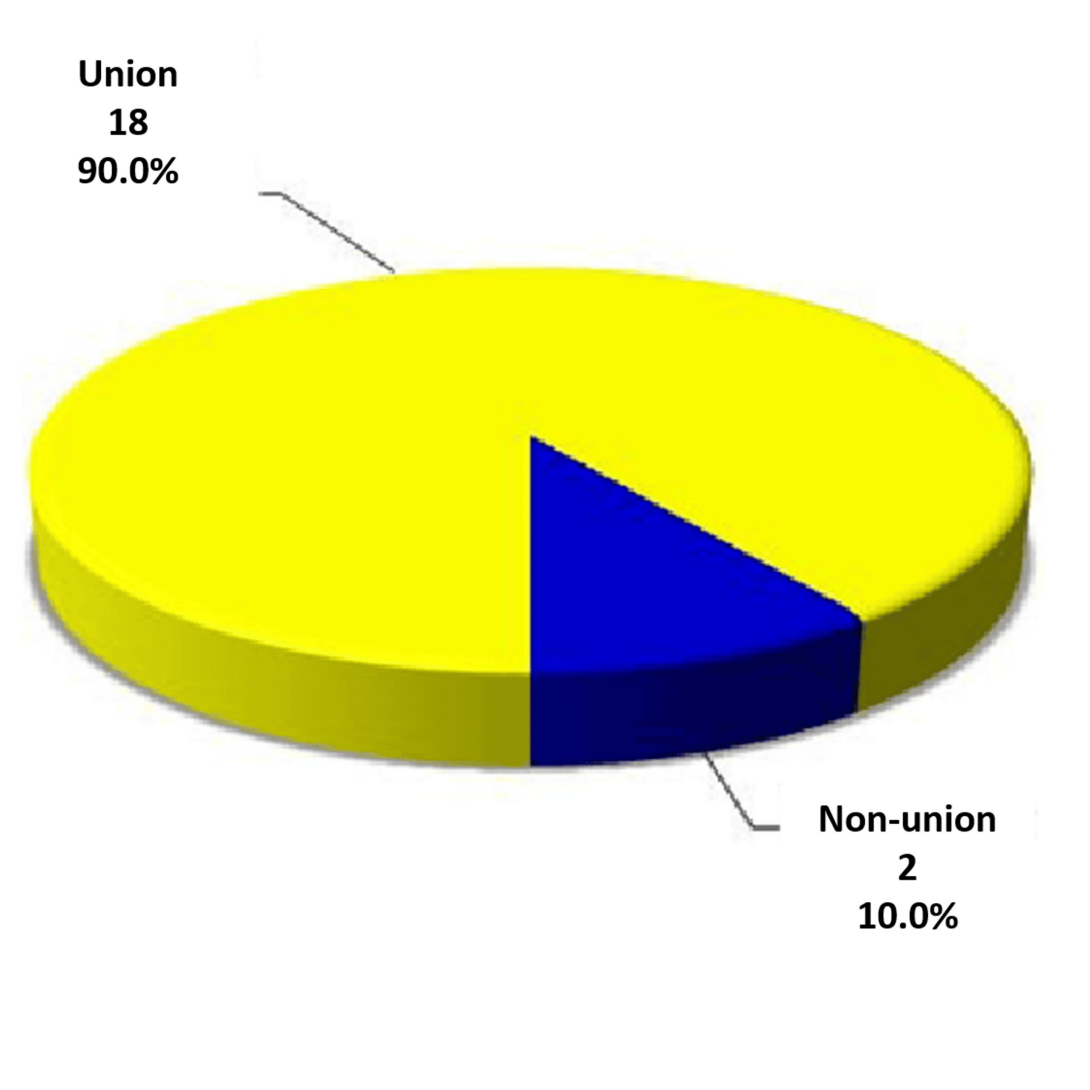
Relationship between healing time and recurrence according to follow-up radiographs

In this study, 18 patients (90%) showed signs of complete union after eight weeks, while two patients (10%) exhibited signs of recurrence within the same period. This indicates that all cases of non-union (100%) were associated with recurrence rather than mechanical failure or patient-related factors. This finding underscores that non-union in this context is likely attributable to the biological behavior of the cyst rather than issues with the graft's mechanical stability or patient compliance (Figure 2).

Correlation between union and MSTS score percentage on follow-up radiographs

According to this study, there was a statistically significant moderate positive correlation between union and MSTS scores (Table 8).

**Table 8:** Relationship between union and MSTS score percentage on follow-up radiographs t: Student t-test; Min: minimum; Max: maximum; MSTS: Musculoskeletal Tumor Society; p-value for association between percentage and follow-up x-rays; *: statistically significant at p ≤ 0.05

Follow-up X-rays	N	Percentage			t	p-value
		Min. – Max.	Mean ± SD.	Median		
Union	18	83.0 – 100.0	93.72 ± 5.37	95.0	5.323^*^	<0.001^*^
Non-union	2	67.0 – 77.0	72.0 ± 7.07	72.0		

Correlation between union and MSTS satisfactory score

The strong correlation between union and MSTS satisfactory scores not only highlights the critical role of achieving complete bone healing, which the use of grafts helps within large cysts, in determining overall patient satisfaction and functional recovery but also indicates that while the MSTS is subjective, it is significant and accurate in determining the functional outcome in this study as shown in Table 9.

**Table 9:** Relationship between percentage and presentation FE: Fisher's exact; Min: minimum; Max: maximum; MSTS: Musculoskeletal Tumor Society;*: statistically significant at p ≤ 0.05

Presentation	N	Percentage			^FE^p-value
		Min. – Max.	Mean ± SD.	Median	
Pain	10	77.0 – 100.0	92.90 ± 7.62	96	0.016*
Swelling	2	87.0 – 94.0	90.50 ± 4.95	90.5	
Pain and swelling	4	67.0 – 100.0	88.50 ± 15.59	93.5	
Fracture	4	87.0 – 97.0	91.75 ± 4.27	91.5	

Correlation between the initial presentation and the MSTS score percentage

**Figure 3:**
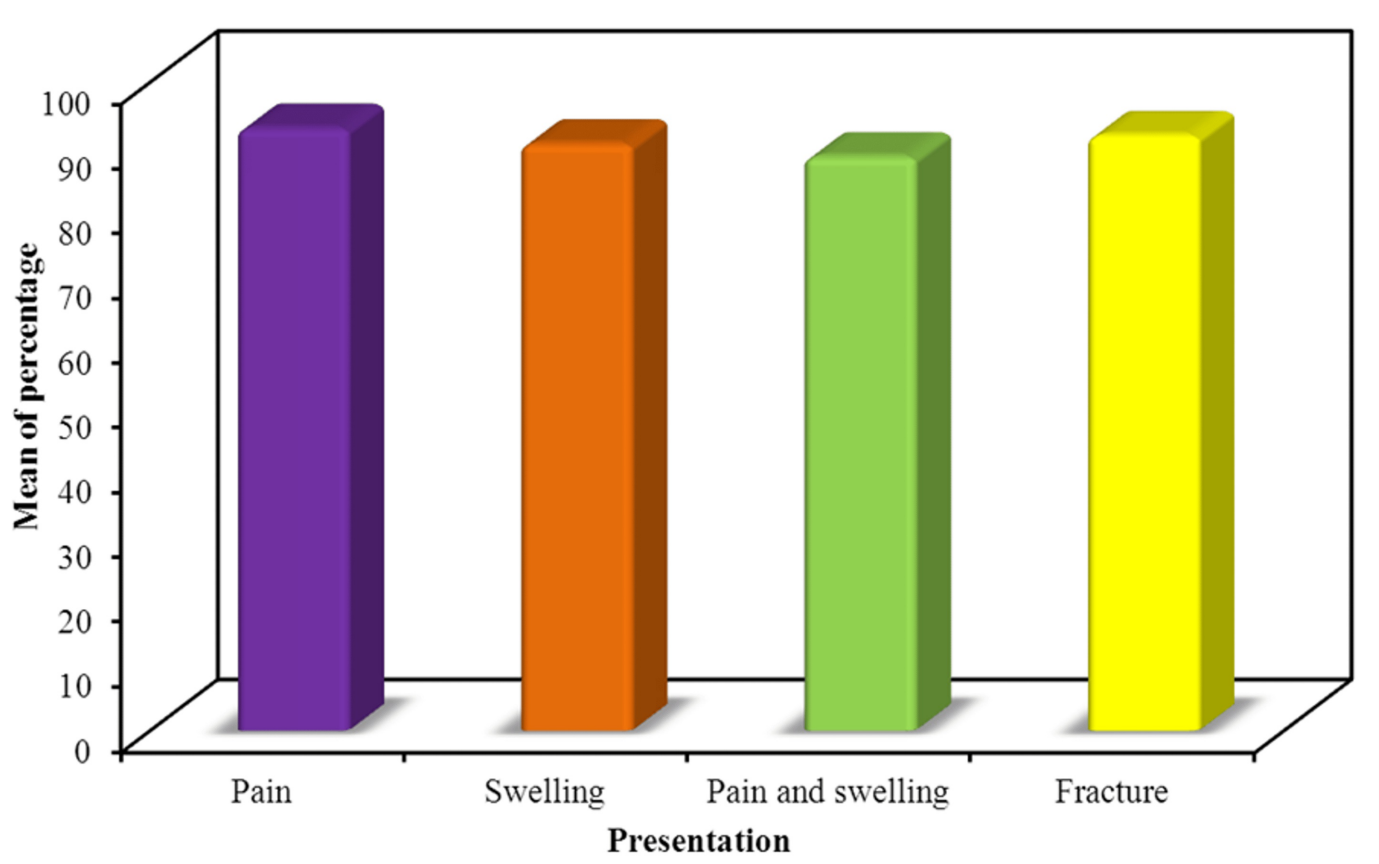
Relationship between percentage and initial presentation

The analysis indicates that there is no statistically significant association between the initial presentation (pain, swelling, pain and swelling, fracture) and the MSTS score percentage (Figure 3). The similarity in mean MSTS scores across different initial presentations suggests that the outcome, as measured by the MSTS score, was not influenced by how the patients initially presented. This finding underscores the notion that the MSTS score outcomes are consistent regardless of the initial clinical symptoms, reinforcing the robustness and reliability of the treatment outcomes across diverse initial presentations, especially fractures.

Preoperative and postoperative radiographic assessment of a patient

**Figure 4:**
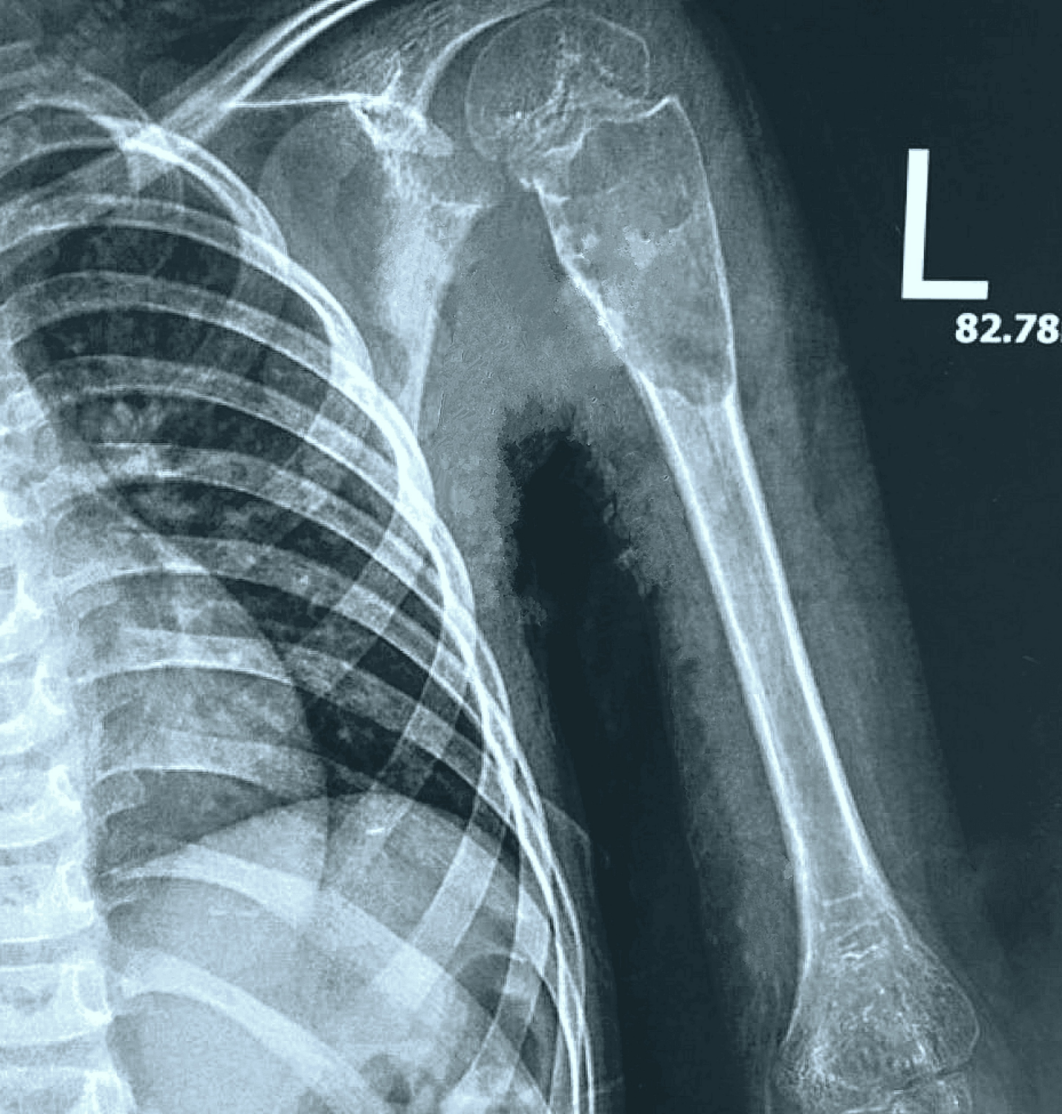
A preoperative radiograph of the left shoulder showing an aneurysmal bone cyst L: left side

The following radiographs represent the preoperative and postoperative images of a nine-year-old patient with left ABC. The preoperative radiograph (Figure 4) shows an osteolytic lesion with cortical thinning.

**Figure 5:**
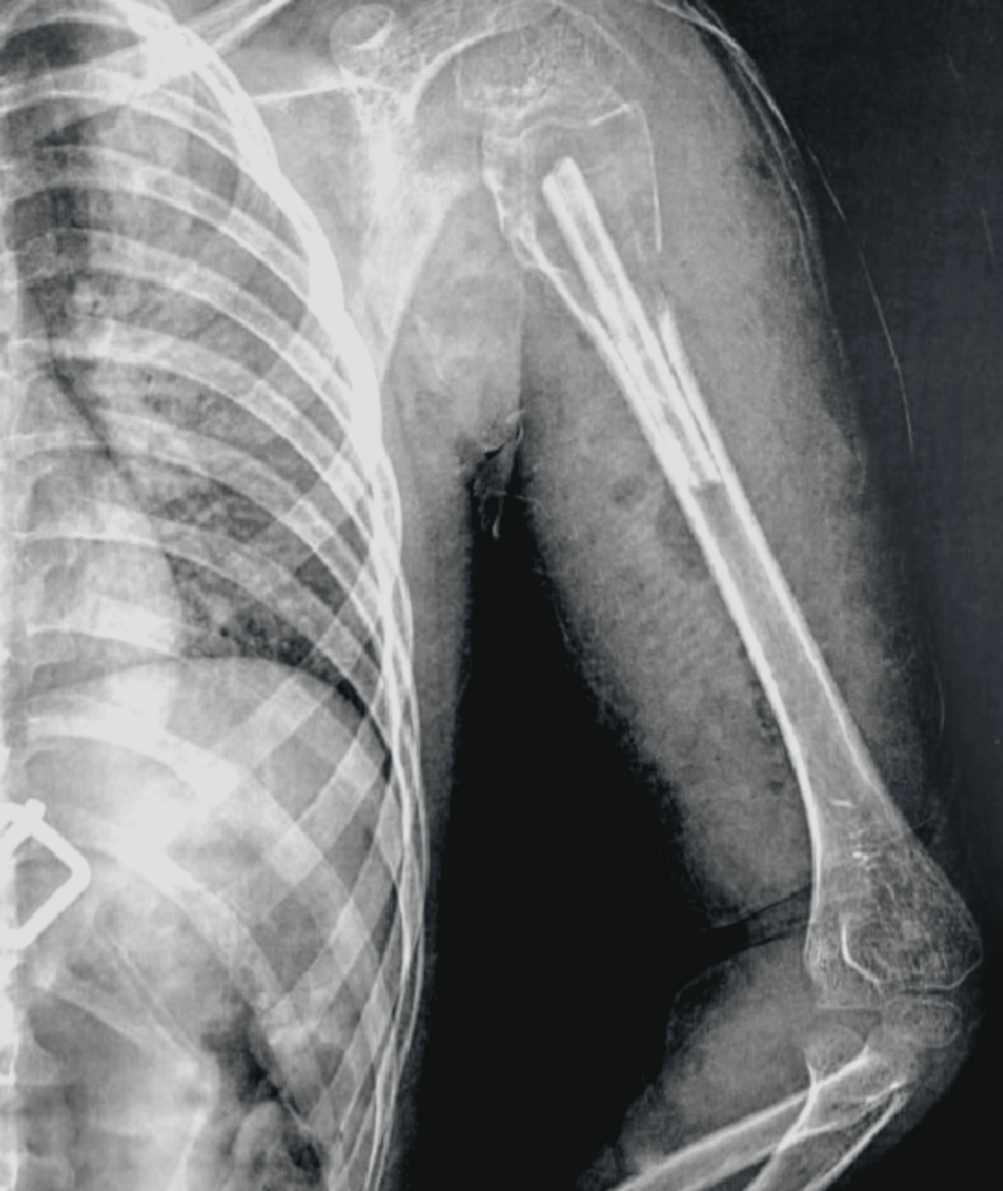
An immediate postoperative radiograph of the left shoulder L: left side

The following radiograph shows the left shoulder immediately postoperatively after curettage of the lesion and impaction of the fibula (Figure 5).

**Figure 6:**
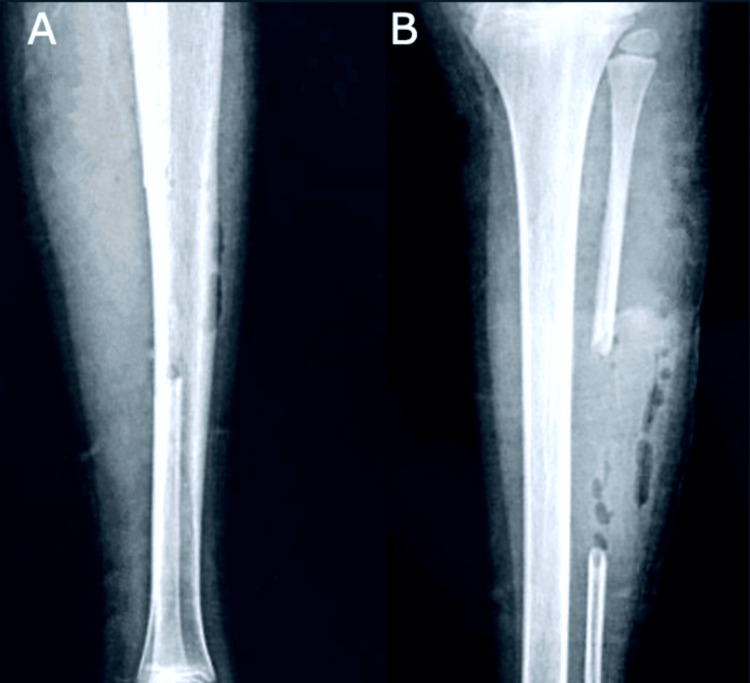
Radiographs of the left leg with the fibula graft taken from the middle third.

The following radiographs of the left leg were obtained after taking the needed graft to preserve the lateral malleolus and its syndesmosis immediately postoperatively (Figure 6).

**Figure 7:**
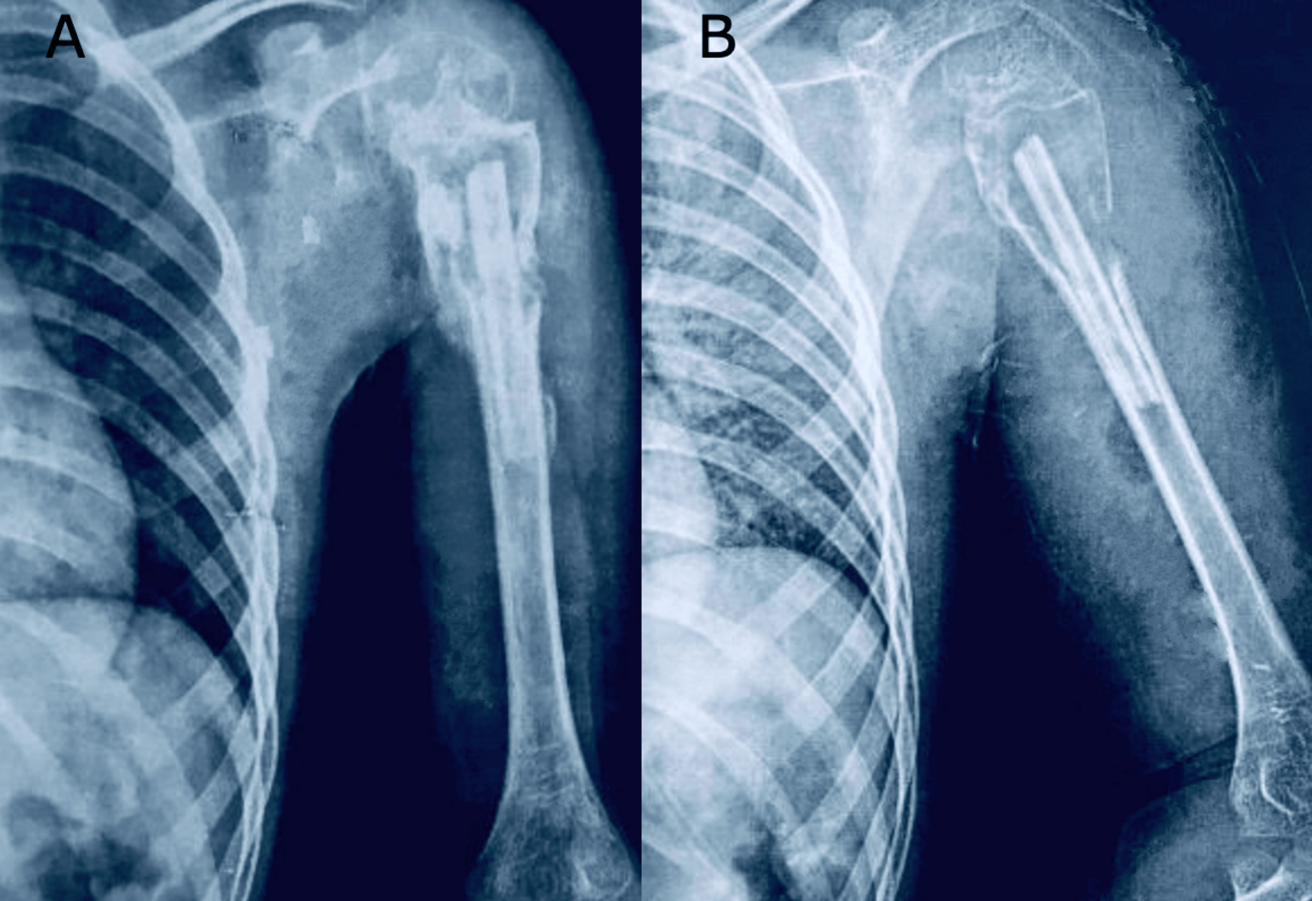
Radiographs obtained three months postoperatively

The following radiographs of the left shoulder show signs of healing of the impacted fibula three months postoperatively (Figure 7).

The following radiograph of the left shoulder six months postoperatively shows the union of the fibula with the proximal humerus with the filling of the cavity (Figure 7). 

**Figure 8:**
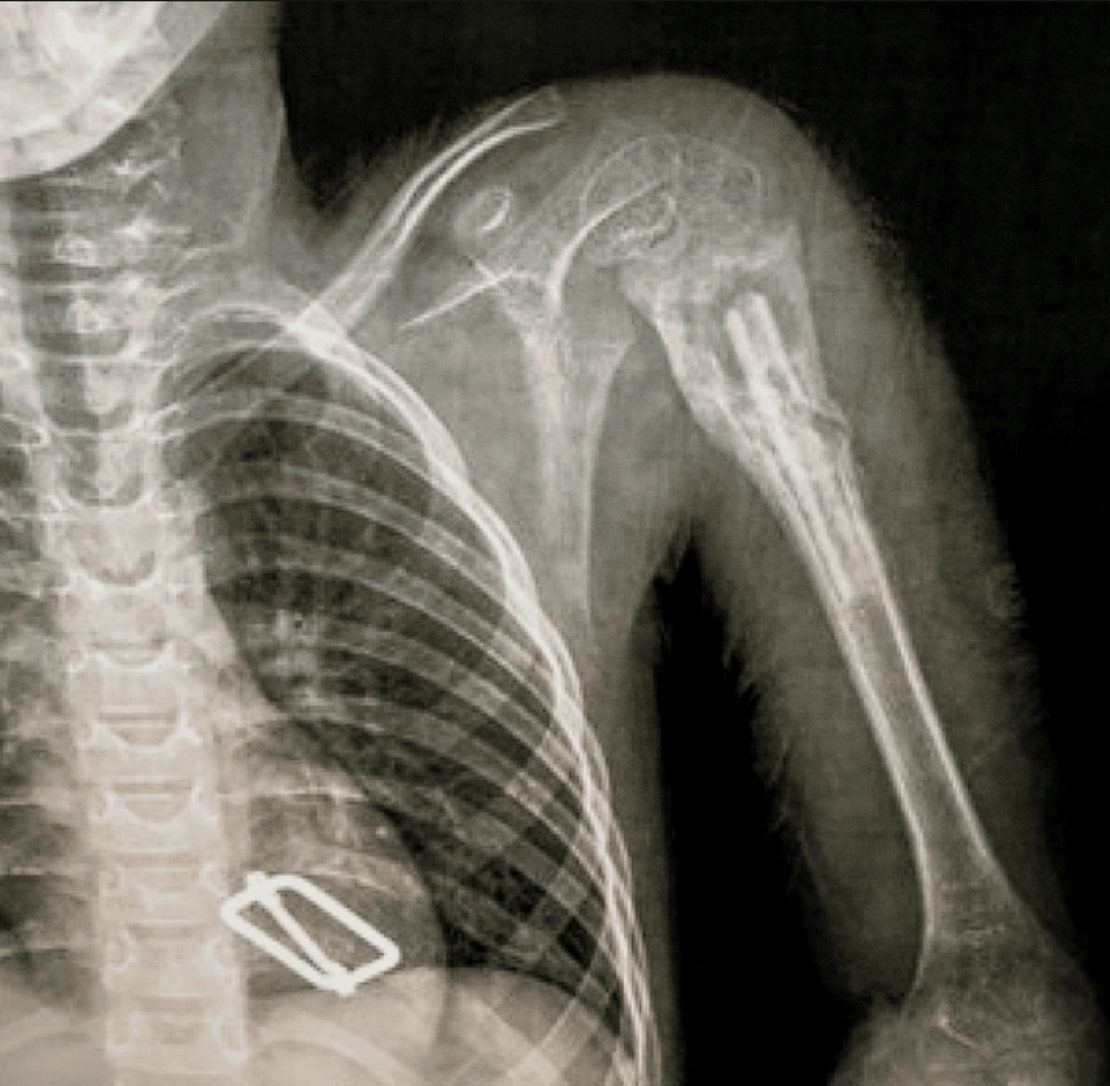
Radiograph taken six months postoperatively

## Discussion

Aneurysmal bone cysts are characterized by their expansive growth pattern and the formation of reactive periosteal new bone, which prevents direct extension into adjacent tissues [1-5]. Due to their weak cortical shell, large ABCs often recur, limiting the effectiveness of internal fixation methods such as plates or screws [1].

In this study, non-vascularized fibular grafts were primarily utilized as intramedullary struts for mechanical stabilization rather than for filling the cyst or preventing recurrence [1]. This approach aimed to facilitate an early range of motion, expedite healing, and minimize morbidity. Fibular grafts provide initial mechanical stability and superior protection against bending forces and collapse compared to iliac bone grafts, with fewer postoperative complications and quicker regeneration potential for future use in cases of recurrence [1].

Literature highlights the complexity of ABC treatment, influenced by factors such as patient age, lesion characteristics, and recurrence risks. Curettage combined with grafting has emerged as a preferred method, demonstrating lower recurrence rates and improved functional outcomes compared to alternative approaches [2-4]. For instance, Campanacci et al. reported a reduced recurrence rate with bone grafts post-curettage compared to cases without grafting [5]. Similarly, studies by Vergel de Dios et al. and Marcove et al. supported the efficacy of curettage with grafting in enhancing patient satisfaction and functional recovery [6-7].

In this study, 70% of patients achieved full function within eight weeks postoperatively, with complete functional recovery observed in all cases by six months. The recurrence rate of 10% underscores the effectiveness of fibular grafts in providing mechanical stability and facilitating early rehabilitation.

Interestingly, this study identified a slight male predominance, contrary to some earlier findings [1, 8, 10, 11]. Given the high risk of recurrence following curettage alone, there has been an exploration into adjunctive therapies, including phenol and cryotherapy, though these modalities present varying success rates and potential complications [9-13].

Hydrogen peroxide (20%) was employed post curettage in this study for its local thermal and oxidizing effects, consistent with previous studies that have highlighted its efficacy and minimal tissue toxicity [8, 10, 14]. Unlike treatments involving radiation or phenol, hydrogen peroxide offers a cost-effective and relatively safe adjunctive option.

The limitations of this study are the small patient cohort, reflecting the rarity of proximal humeral ABCs, and also stringent inclusion criteria necessitating a cyst length-to-width ratio of at least two. Smaller cysts typically heal spontaneously without requiring fixation. Additionally, focusing on a specific anatomical site in the upper limb and relying on the MSTS upper limb score, while widely accepted for evaluating clinical outcomes in orthopedic oncology patients, introduces subjectivity that may lead to variability in patient-reported outcomes.

## Conclusions

In conclusion, utilizing an impacted, non-vascularized autogenous fibular graft for treating proximal humeral ABCs proves highly effective in providing mechanical stability and facilitating an early return to normal function, often before the complete osseous filling of the defect. The fibular graft's mechanical properties uphold the structural integrity of the proximal humerus, enabling patients to swiftly resume daily activities with minimal disruption. Furthermore, the need for cavity refilling post-curettage is eliminated, as the cavities undergo spontaneous healing within a reasonable timeframe. These findings highlight the graft's practical value in clinical settings, offering a reliable, efficient, and less invasive approach to managing this condition. Ultimately, this approach contributes to enhanced patient outcomes and alleviates healthcare burdens.
